# Development of novel rectal/uterine clamping device

**DOI:** 10.1038/s41598-024-75103-y

**Published:** 2024-10-03

**Authors:** Shoichiro Urabe, Taishi Hata, Eiji Kobayashi, Yuji Ishii, Yuki Ushimaru, Mitsunobu Takeda, Yuki Sekido, Tsuyoshi Hata, Atsushi Hamabe, Takayuki Ogino, Norikatsu Miyoshi, Mamoru Uemura, Hirofumi Yamamoto, Yuichiro Doki, Hidetoshi Eguchi, Kiyokazu Nakajima

**Affiliations:** 1 Department of Next Generation Endoscopic Intervention (Project ENGINE), Osaka University Graduate School of Medicine, Suite 0802, BioSystems Bldg., 1-3, Yamadaoka, Osaka, 565-0871 Osaka Japan; 2 Department of Gastroenterological Surgery, Osaka University Graduate School of Medicine, Osaka, Japan; 3 Obstetrics and Gynecology, Oita University Faculty of Medicine, Oita, Japan; 4 CASTEM Co, Ltd, Hiroshima, Japan

**Keywords:** Clamping device, Clamper, Laparoscopic surgery, Bursting pressure, micro-CT, Gastrointestinal cancer, Gynaecological cancer, Preclinical research

## Abstract

We developed a novel clamping device for laparoscopic surgery, free from conventional pinch structure, capable of uniformly occluding any ductal organ. This study aimed to evaluate performance of the new clamper compared to the pinch-type clamper. The new clamper consists of two metal bars with ties at each end, which enables parallel clamping. A resected porcine stomach was used, with an infusion tube at the anal end to increase intra-luminal pressure. The oral side of the stomach was clamped with either the new clamper or the pinch-type clamper, and their performances were evaluated in qualitative and semi-quantitative manner. Qualitative evaluation involved imaging the clamping site at intra-gastric pressures from 0 to 15 mmHg using microfocus computed tomography. The new clamper showed no gap even under increased intra-luminal pressure, while the pinch-type clamper showed a gap on the distal side. Quantitative evaluation measured bursting pressure under continuous air insufflation. Air leakages were observed in the new clamper at higher intra-luminal pressures than in the pinch-type clamper (46.1 mmHg vs. 13.6 mmHg, *P* < 0.01). Our new clamping device showed superior performance in preclinical setting compared to the conventional pinch-type clamper. We are currently working on its design freezing and aiming for early commercialization.

**Supplementary Information:**

The online version contains supplementary material available at 10.1038/s41598-024-75103-y.

## Introduction

**Fig. 1:**
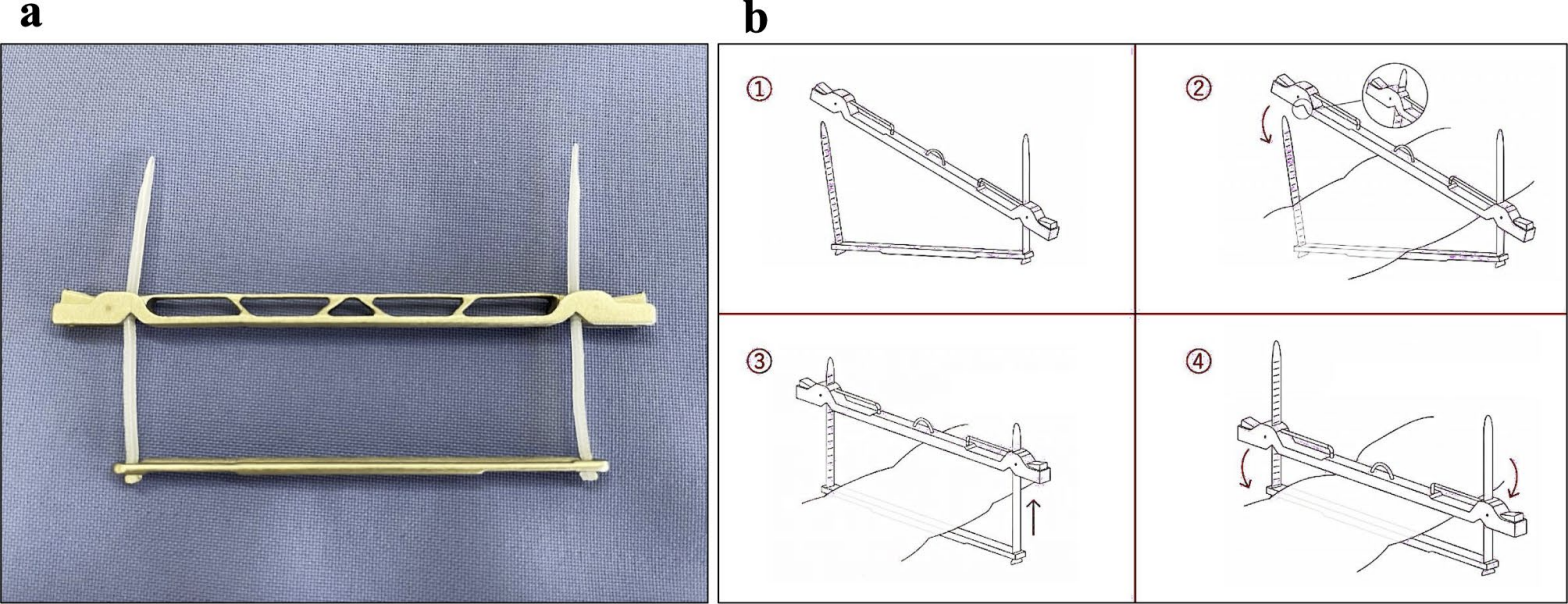
Development of the New Clamper. (**a**) Design of the new clamper. (**b**) How to operate the new clamper.

Clampers are surgical devices widely used in various surgical fields to temporarily occlude ductal organs. In gastrointestinal surgery, for example, clampers are used to occlude the rectum during rectal washout in sigmoid colon and rectal cancer surgery^1–5^. In gynecology, for instant, clampers are used in cervical cancer surgery to occlude vaginal tract to prevent cancer spillage out of the resected vaginal stump^6^. There are various shapes of clampers, but most of them used in laparoscopic surgery have a pinch-type structure with limited opening angle and uneven clamping force, which can potentially cause clinical problems due to suboptimal clamping in the case of thick ductal organs^7^. From these backgrounds, we started to develop a new clamper for laparoscopic surgery that can optimally occlude any shape of ductal organs. We conceived of a clamper with a totally new mechanism and created a prototype (Fig. 1). The purpose of this study was to assess the usability of our new clamper in in-vivo setting, and to evaluate the performance of the new clamper compared with conventional pinch-type clamper qualitatively and semi-quantitatively in ex vivo experiments.

## Results

### In-vivo experiment

**Fig. 2:**
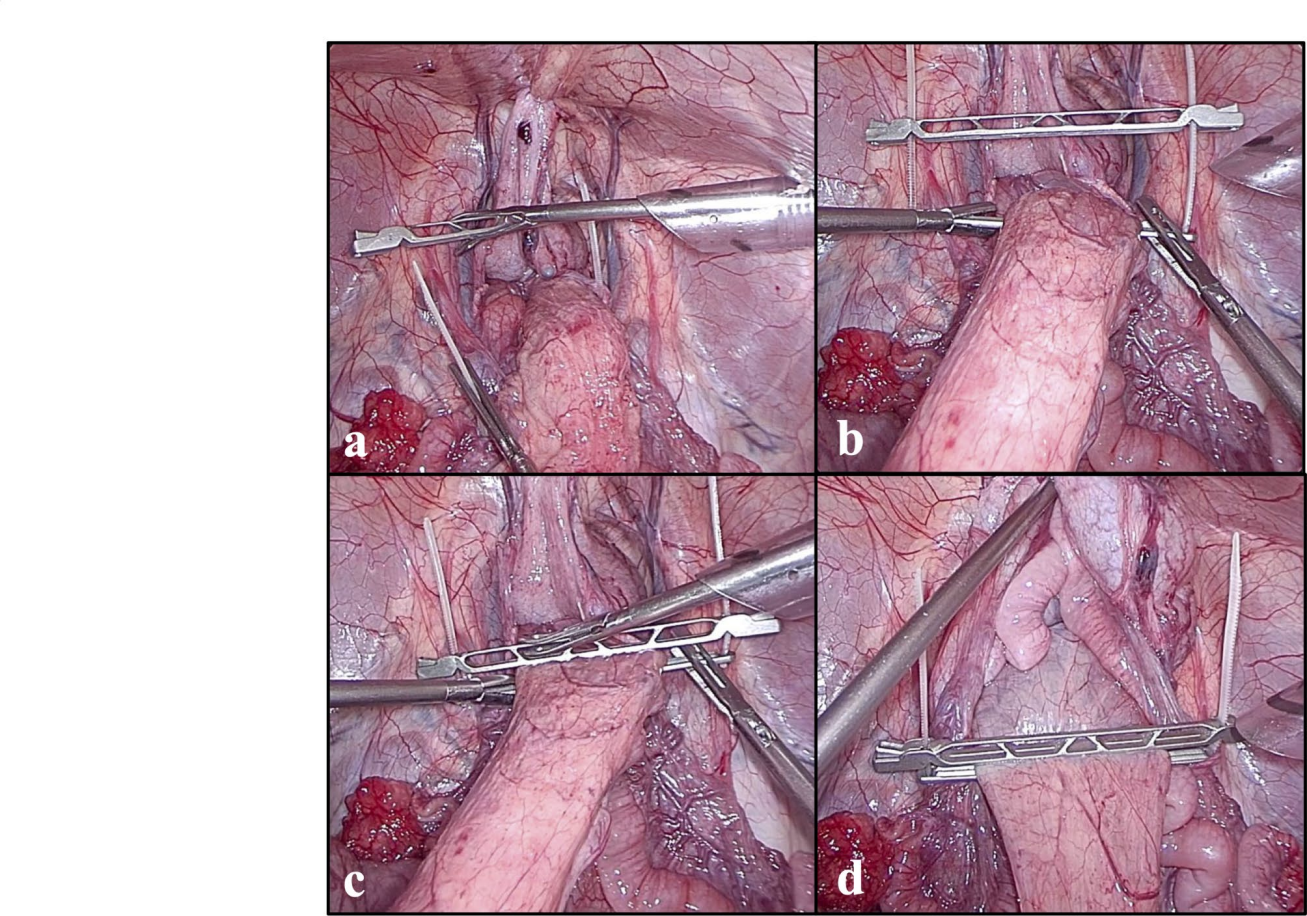
laparoscopic rectal clamping and rectal washout in the pig body. (**a**) Encircle the rectum with the clamper and close the tie. (**b**) Move the clamper to the optimum position. (**c**) Alternately close the clamper from left to right until completely closed. **d**, Performed rectal washout.

**Fig. 3:**
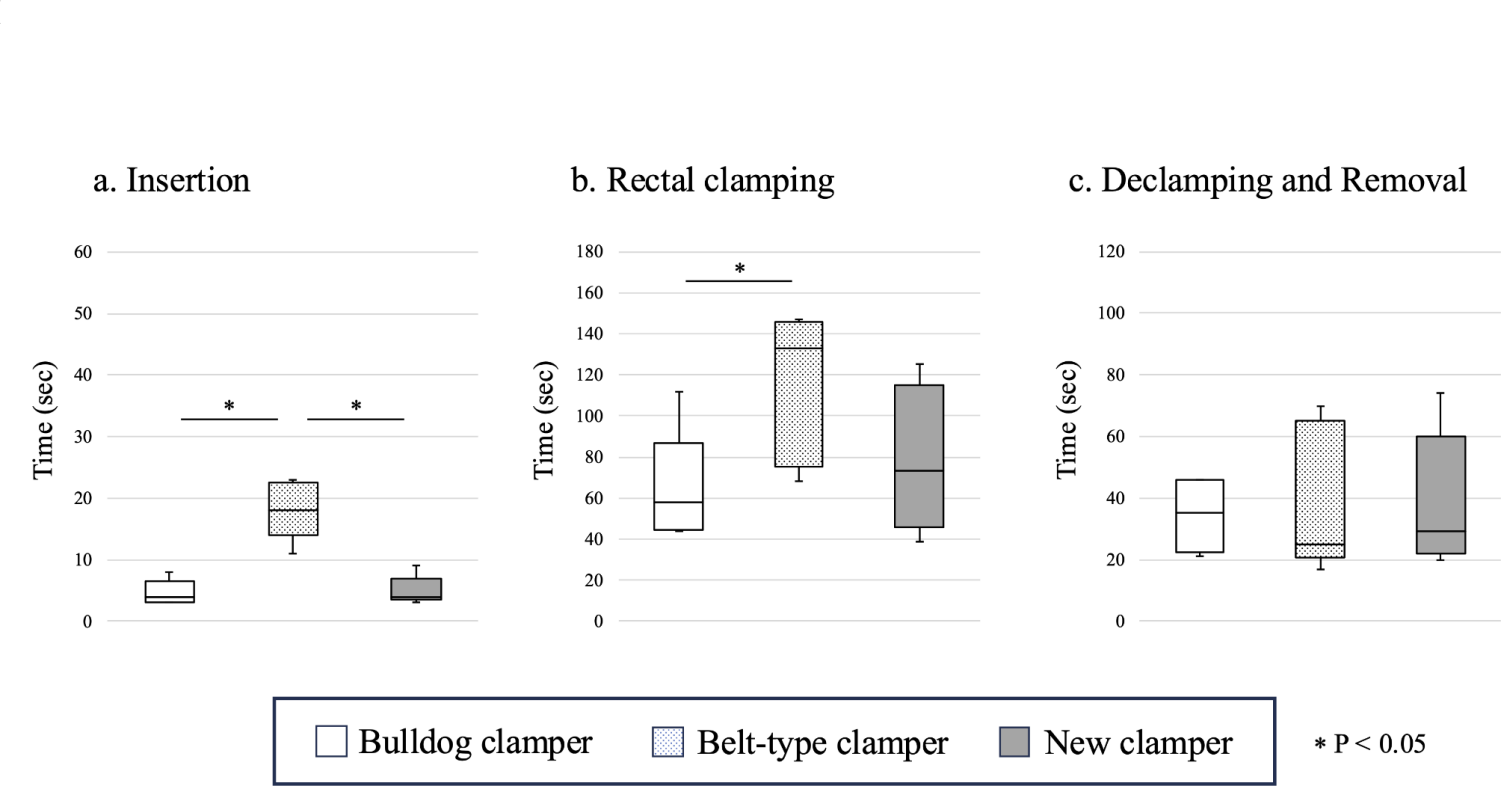
Operation time for porcine rectal clamping.(**a**) Time required for insertion through the trocar (4.0 Sect. [3.0-6.5] vs. 18.0 Sect. [14.0-22.5] vs. 4.0 Sect. [3.5-7.0]). (**b**) Time required for rectal clamping (58.0 Sect. [44.5–87.0] vs. 133.0 Sect. [75.5–146.0] vs. 73.0 Sect. [45.5–115.0]). (**c**) Time required for declamping and removal through the trocar (35.0 Sect. [22.5–46.0] vs. 25.0 Sect. [20.5–65.0] vs. 29.0 Sect. [22.0–60.0]).

The new clamper was successfully passed into abdomen through a standard 12 mm laparoscopic trocar (ENDOPATH XCEL bladeless trocar, Ethicon Endo-Surgery, Inc., OH, USA) and was able to be manipulated with standard laparoscopic forceps (CLICKLINE Forceps, KARL STORZ SE & Co. KG, Tuttlingen, Germany). In this experiment, our new clamper occluded the porcine rectum with the expected behavior. After occlusion we successfully completed rectal washout procedure, with the clamper placed in-situ without any slippage (Fig. 2). The time required for insertion through the trocar, rectal clamping, and declamping and removal through the trocar were compared between the bulldog clamper, the belt-type clamper and our new clamper. The results were as follows: insertion time (4.0 Sect. [3.0-6.5] vs. 18.0 Sect. [14.0-22.5] vs. 4.0 Sect. [3.5-7.0]), rectal clamping time (58.0 Sect. [44.5–87.0] vs. 133.0 Sect. [75.5–146.0] vs. 73.0 Sect. [45.5–115.0]), and the time of declamping and removal (35.0 Sect. [22.5–46.0] vs. 25.0 Sect. [20.5–65.0] vs. 29.0 Sect. [22.0–60.0]) for the bulldog clamper, the belt-type clamper and the new clamper, respectively (Fig. 3). No statistically significant differences were observed in the time required for each operation between the bulldog clamper and the new clamper. However, the belt-type clamper took longer for insertion and rectal clamping compared to the other devices.

## Ex-vivo experiments

### Experiment 1 -qualitative evaluation-

**Fig. 4:**
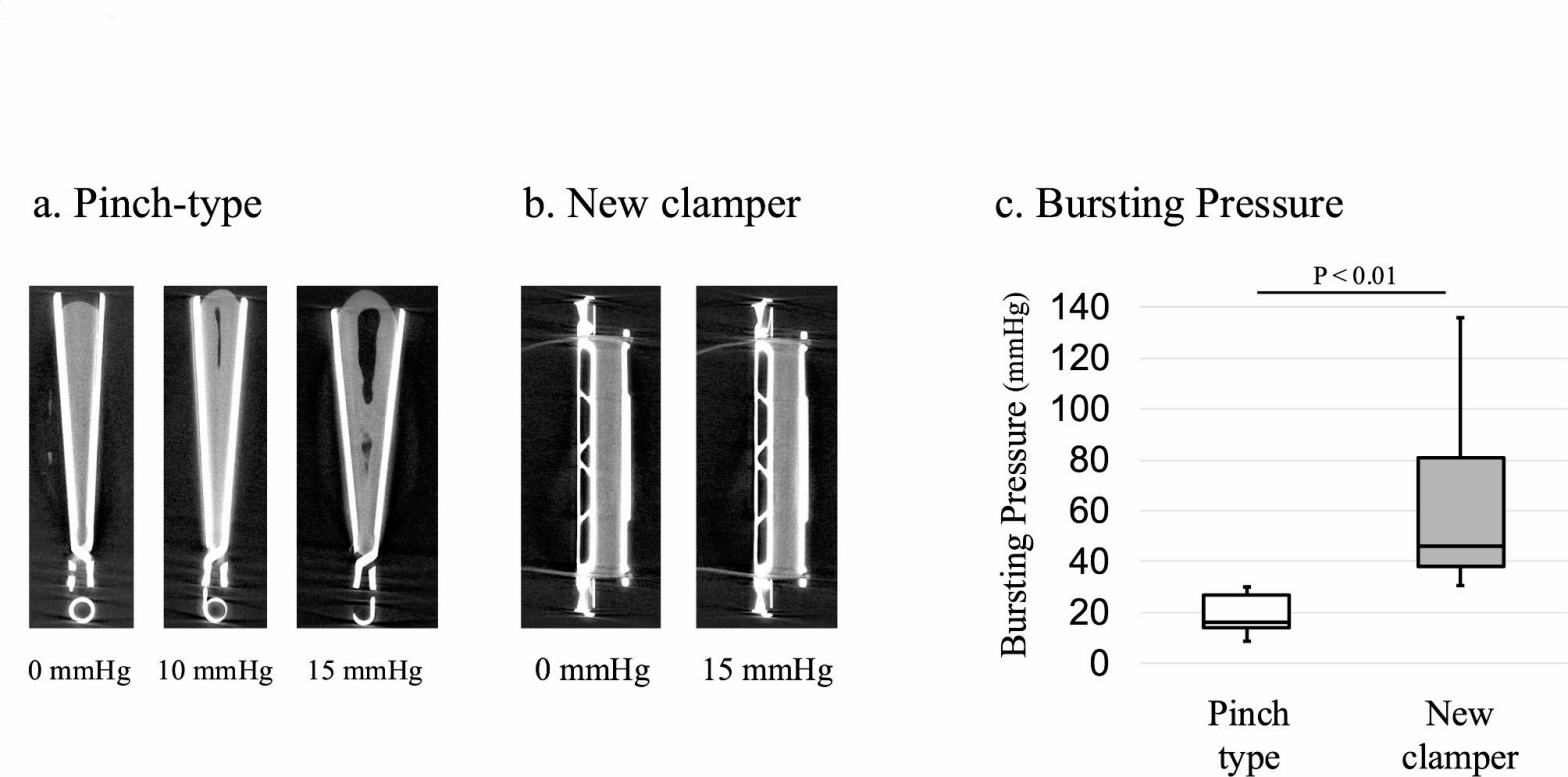
Results of Ex-vivo experiments (qualitative and quantitative evaluation). (**a**) Gaps gradually enlarged due to increased intra-gastric pressure. (**b**) Uniformly occluded even under increased intragastric pressure. (**c**) The new clamper showed significantly higher bursting pressure than pinch-type clamper. (46.1 mmHg [37.8–80.8] vs. 13.6 mmHg [11.8–15.4], *P* < 0.01)

In the specimens with the pinch-type clamper, the clamping site expanded in a wedge-like shape from the hinge side to the distal side, and a gap was observed on the distal side when the intra-luminal pressure reached 10 mmHg, and the gap enlarged further at 15 mmHg of intra-luminal pressure (Fig. 4a). In the specimens with the new clamper, the clamping site was closed uniformly, and no gap was observed even when the intra-luminal pressure increased to 15 mmHg (Fig. 4b).

## Experiment 2 -quantitative evaluation-

### Bursting experiment

Air leakages were observed in the new clamper at statistically significantly higher intra-luminal pressures than in the pinch-type clamper. (46.1 mmHg [37.8–80.8] vs. 13.6 mmHg [11.8–15.4], *P* < 0.01) (Fig. 4c).

In all cases, both with the pinch-type and the new clampers, air leakage occurred through gaps in the clamper due to increased intra-luminal pressure.

## Discussion

Clamping devices are widely used in variety of surgical subspecialities to temporarily occlude ductal organs. In gastrointestinal surgery, rectal washout is a commonly employed technique, particularly during anastomosis for rectal cancer surgery, with the aim of preventing local and anastomotic recurrence^1–5^. Most of the clampers used in laparoscopic surgery have a pinch structure, but it is sometimes technically difficult to occlude thick rectum with pinch-type clampers due to limited opening angle and uneven clamping force. In gynecology, the LACC trial reported in 2018 that laparoscopic surgery for cervical cancer is inferior to open surgery in DFS and OS^6^. Subsequently, several reports have shown that laparoscopic surgery for cervical cancer was associated with an increased risk of recurrence and death compared to open surgery^8–10^. Following these report, laparoscopic surgery for cervical cancer has been restricted worldwide. In laparoscopic surgery for cervical cancer, intracorporeal colpotomy under CO2 pneumoperitoneum might be related to the risk of intraperitoneal tumor spillage, which is promoted by the circulating CO2^6,8–13^. To date, various attempts have been made to prevent cancer cells from being exposed to circulating CO2 during intracorporeal colpotomy^14–19^. However, there are no devices capable of easily and optimally occluding the thick vaginal tract laparoscopically. For these reasons, we identified a clinical need for a new clamping device that could be properly applied with a simple manipulation.

The features of the new clamper included: (1) the capability to be inserted and removed via standard laparoscopic trocars, (2) applicability to the appropriate position, (3) ease of clamping, (4) non-entanglement with surrounding tissue in the process of application, (5) parallel and uniform clamping force, and (6) no requirement of additional and/or special instruments.

We attempted to perform laparoscopic rectal clamping in the porcine abdomen using the prototype of our new clamper. The new clamper was passed into the abdomen through a standard 12 mm trocar and was manipulated using only standard laparoscopic forceps. We then attempted rectal washout. During the procedure, our new clamper did not slip from its original position, and after the procedure, it was easily released and removed via the trocar. We also compared the time required for the clamping operation between the conventional pinch-type clampers (the bulldog and the belt-type clamper) and the new clamper. There was no statistically significant difference in operation time between our new device and the bulldog clamper, but the new device required significantly less time for insertion compared to the belt-type clamper. These results showed that our new clamper performed as expected in laparoscopic surgery.

We also evaluated the performance of the new clamper in comparison with a pinch-type clamper that is commonly used in laparoscopic surgery using ex-vivo models. All surgeons should use clamping devices that are sufficiently long to fully occlude the target organ, and under such conditions, all existing clampers are clinically adequate. However, in clinical practice, the target organ may become enlarged due to inflammation or other factors, and existing laparoscopic clampers may be suboptimal for clamping, potentially leading to clinical complications. Therefore, in this study, we evaluated each clamper by using thick porcine stomachs to reproduce situations in which clamping is difficult with existing clampers. We adopted micro-CT as the method of qualitative evaluation and bursting pressure as the method of quantitative evaluation. Industrial micro-CT has traditionally been used in the engineering industry as a method of structural non-destructive analysis of precise machines, but it is not used as a general technique in life science fields due to the need for long exposure times and high level of radiation^20,21^. In recent years, several studies reported the usefulness of micro-CT in the evaluation of ex-vivo human tissue, and our laboratory has also reported the usefulness of industrial micro-CT as a diagnostic aid in surgical treatment^22–24^. To our knowledge, this study is the first report of a micro-CT evaluation of clamping devices. In this study, micro-CT images of a porcine stomach clamped with a pinch-type clamper showed a gap at the distal part of the clamper due to increased intra-gastric pressure. This result is consistent with previous reports that pinch-type clampers have weak clamping force at the distal part^7,25,26^. Whereas CT images of the specimen with the new clamper showed that the porcine stomach was uniformly occluded without gaps, and remained the condition even under high intra-luminal pressure.

The bursting pressure experiment is a traditional method for assessing the strength of anastomotic sites, and there are a number of reports using this method^27–32^. Some studies have also adapted the bursting pressure experiment to measure the strength of clampers^7,25,26^. Therefore, in this study, we applied this method for the quantitative evaluation of clampers. In the bursting pressure experiments, the new clamper showed higher bursting pressures than the pinch-type clamper, which indicates that our new clamper has higher clamping strength than the conventional pinch-type clampers.

The belt-type clamper resembles our new device in that both have a similar mechanism of occluding bars by tightening ties. However, there are significant differences in their structures and functions. First, we categorized the belt-type clamper as a type of pinch-type clamper. It has a hinge structure on one side, causing the bars to move in a fan shape, whereas our new device features movable parts on both the left and right sides, allowing for parallel movement. Although this difference may not be significant when clamping thin organs, it becomes more pronounced when clamping thick organs, such as a swollen rectum or vaginal tract. Supplementary Fig. 1a illustrates the structural differences between the two devices. Second, the belt-type clamper cannot be securely fixed until the upper and lower bars are fully occluded. In contrast, our new device allows for adjusting the degree of occlusion depending on the thickness of the clamped object. We were unable to perform a quantitative evaluation with the belt-type clamper because it could not fully occlude the pyloric part of the porcine stomach, which we defined as the clamping site in ex-vivo experiment. However, we have provided a reference image in Supplementary Fig. 1b, which shows the cardiac part of the porcine stomach (thinner than the pyloric part) clamped with the belt-type clamper and qualitatively evaluated using micro-CT. From the CT image, it can be observed that the bars of the belt-type clamper spread from the hinge side to the distal side, and the bars are deformed due to the strong force required to clamp thick organs. From these observations, our new device is superior in that it can easily and uniformly occlude ductal organs of any thickness.

The results of this study showed the usefulness of the new clamper, but several limitations remain. First, these experiments were conducted on porcine intestines, not human organs, and the porcine uterus was not used because its organ structure is substantially different from that of a human uterus. Second, there is no established method for evaluating the function of clampers. Although the new clamper showed superiority over the pinch-type clamper in the qualitative and quantitative evaluation experiments in this study, it is still unclear whether these results correlate with clinical outcomes such as anastomotic recurrence rate after cancer surgery. We will proceed to commercialize the new clamper and evaluate it in clinical settings to determine its clinical outcomes.

We developed a new clamping device free from pinch structure, and assessed its usability and performance in comparison with the conventional clampers, in in-vivo and ex-vivo settings. The new clamper was easy to use in laparoscopic surgery and occluded the porcine intestinal tract in a uniform fashion, and showed higher performance than the conventional pinch-type clamper in ex-vivo experiments. The device is now in the final process for mass production and commercialization.

## Materials and methods

### Clampers

**Fig. 5:**
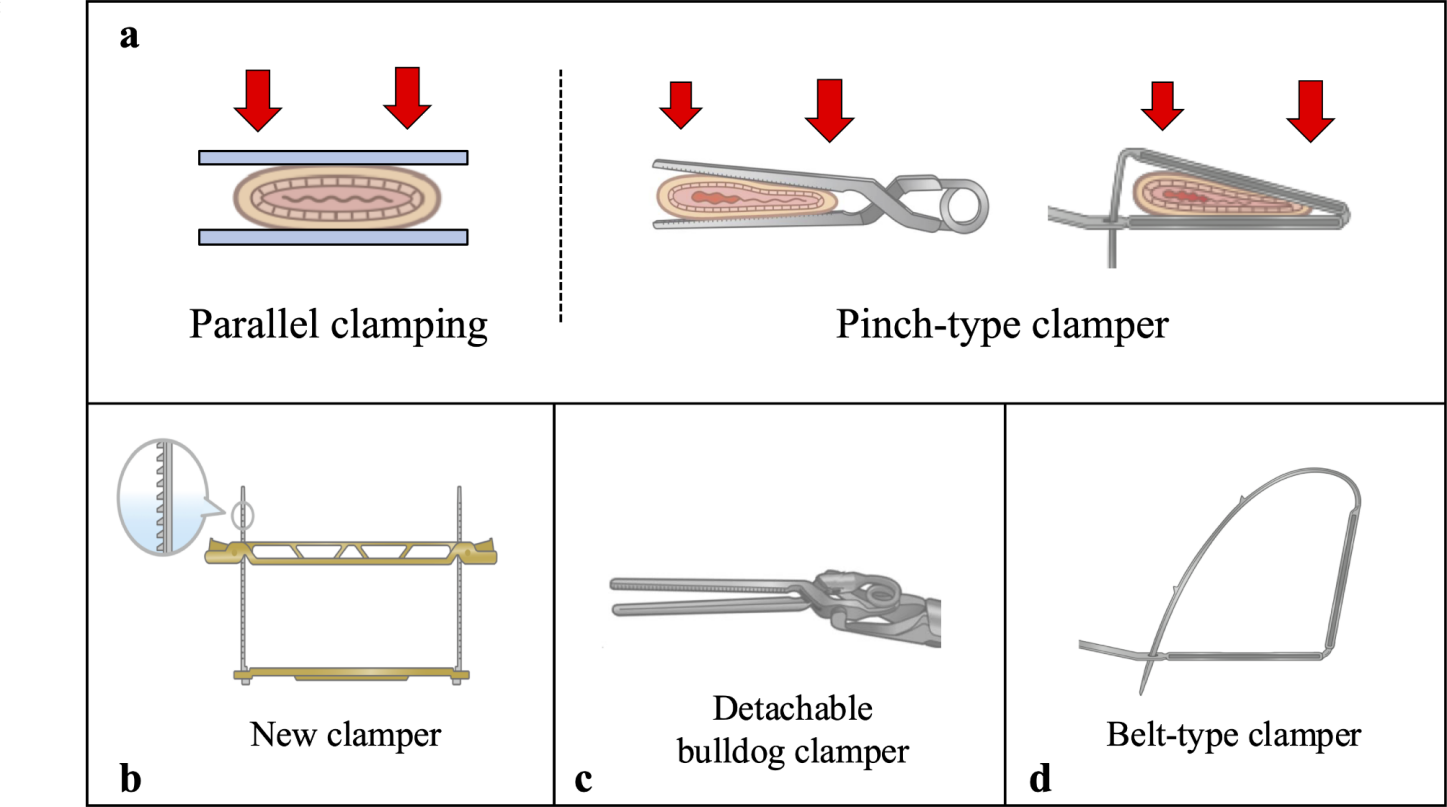
Structure of Clampers. (**a**) Difference of clamping force. (**b**) Structure of the new clamper. (**c**) Schema of Detachable bulldog clamper. (**d**) Schema of Belt-type clamper.

Figure 1 shows our newly developed clamping device (Tied Bar Clamper, CASTEM Co, Ltd, Hiroshima, Japan). We hypothesized that pinch-type clampers would fail to completely occlude thick ductal organs due to uneven clamping force, whereas parallel clamping would be able to optimally occlude any thick ductal organs (Fig. 5a). Based on this hypothesis, we conceived of a clamper with a totally new mechanism. The new clamper has a structure consisting of two metal bars with two ties at each end (Fig. 5b). The bars are made of AISI 316 stainless steel, and the ties are made of nylon resin (PA66). We produced a prototype of this clamper by metal 3D printer (TruPrint 1000, TRUMPF Co., Ltd., Ditzingen, Germany) and used it in the following study. The detachable bulldog clamper (Premium DeBakey Clamp, C70S, HEIWA Medical Instruments Co., Ltd., Yamaguchi, Japan) and the belt-type clamper (Gut Clamper, Kobe Biomedix Co., Ltd., Kobe, Japan) were used as pinch-type clampers for comparison (Fig. 5c and d).

### In-vivo experiment

A three-month-old female pig, weighing 35 kg, was used under general anesthesia. This study was approved by the Institutional Animal Care and Use Committee of IVTeC Co. Ltd. (Animal Welfare Committee, approval number: IVT 23 − 21). To test the usability, we performed laparoscopic rectal clamping using the new clamper in the porcine abdomen and after occluding the porcine rectum, we performed rectal washout by injecting 100 ml of saline solution through the porcine anus. In an experiment to compare operation times between the conventional pinch-type clampers and the new clamper, five gastrointestinal surgeons performed the following tasks with each device: (1) insertion through the trocar, (2) rectal clamping, and (3) declamping and removal through the trocar, and the time required for each operation was measured (*n* = 5). The detachable bulldog clamper was manipulated with dedicated instruments: the removal forceps (premium angle forceps, RM1, HEIWA Medical Instruments Co., Ltd., Yamaguchi, Japan) and the applier forceps (premium angle forceps, B60, HEIWA Medical Instruments Co., Ltd., Yamaguchi, Japan). Animals were euthanized at the end of experiment using rapid intravenous administration (1 ml/kg) of saturated potassium chloride solution under deep anesthesia.

### Ex-vivo experiments

**Fig. 6:**
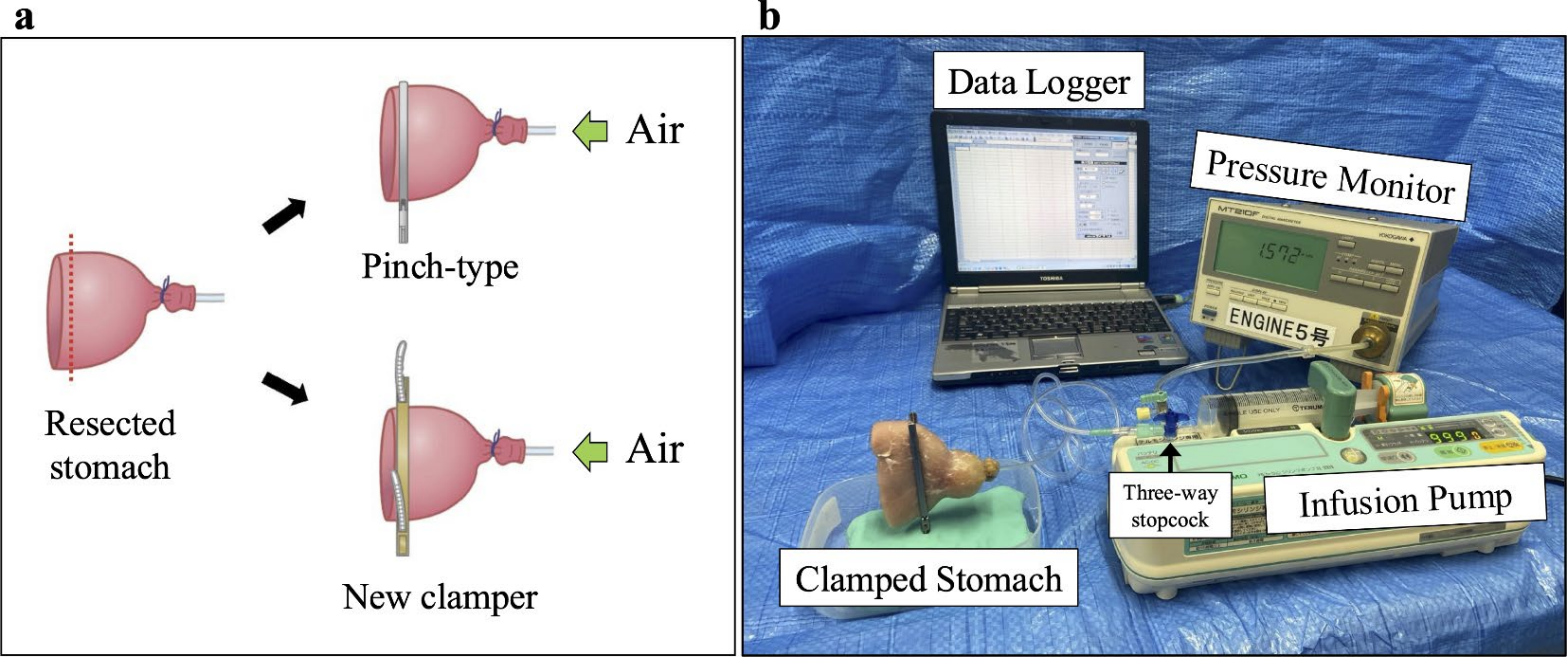
Schema of ex vivo experiments. (**a**) Preparation of the resected stomach model. (**b**) Set up of the bursting pressure experiments.

We prepared the pyloric side of the resected porcine stomach and placed a 2.5 mm infusion tube (JMS extension tube, JV-ND2050LE, JMS Co., Ltd, Tokyo, Japan) on the duodenal side and tightly fastened it (Fig. 6a). The oral side of the resected porcine stomach was left open for occlusion with a clamper. To ensure consistency in the clamping position for each clamper, we marked a site near the pylorus. An automatic infusion pump (Terufusion, TE-332 S, Terumo Corp., Tokyo, Japan) as an insufflator and a digital manometer (MT210F, Yokogawa Test & Measurement Corporation, Tokyo, Japan) were connected to the infusion tube using a three-way stopcock. Pressure measurements (mmHg) were taken and recorded on a dedicated personal computer (Fig. 6b).

### Experiment 1 -qualitative evaluation-

The oral side of the prepared stomach was closed with either the pinch-type clamper or the new clamper and was insufflated with room air from the opposite side to raise the intra-luminal pressure. The images of the clamping site at the intra-luminal pressure of 0 to 15 mmHg, were obtained in non-destructive fashion using industrial microfocus computed tomography (micro-CT) (MCT225, NIKON Solutions Co., Ltd., Tokyo, Japan) and compared qualitatively. For this qualitative evaluation, two resected porcine stomachs were used, and the experiment was repeated three times for each of the pinch-type clamper and the new clamper.

### Experiment 2 -quantitative evaluation-

The oral side of the prepared stomach was closed with either the pinch-type clamper (*n* = 10) or the new clamper (*n* = 10) and was insufflated with room air from the opposite side with a rate of 0.25 mL/s (900 mL/h). The bursting pressure (the maximum intra-luminal pressure when the air leak occurred) and the bursting point (the site where the air leak occurred) were recorded and compared by real-time pressure monitoring under continuous insufflation.

### Statistical analysis

Statistical analyses were performed using a dedicated statistical software package (JMP Pro, version 17.2.0, SAS Institute, Inc., Cary, NC, USA) on a universal personal computer. Continuous data are expressed as median [interquartile range], unless otherwise indicated. Subsequent comparisons between the groups were made by Mann-Whitney U test. A *p*-value of < 0.05 was considered statistically significant.

## Electronic supplementary material

Below is the link to the electronic supplementary material.

Supplementary Material 1

Supplementary Material 2

Supplementary Material 3

## Data Availability

The datasets generated during and/or analysed during the current study are available from the corresponding author on reasonable request.
