# Supplementary figures and images for: Development of novel rectal/uterine clamping device
Source: Sci Rep. 2024 Oct 3;14:22960. doi: 10.1038/s41598-024-75103-y (PMC11450202; doi:10.1038/s41598-024-75103-y)

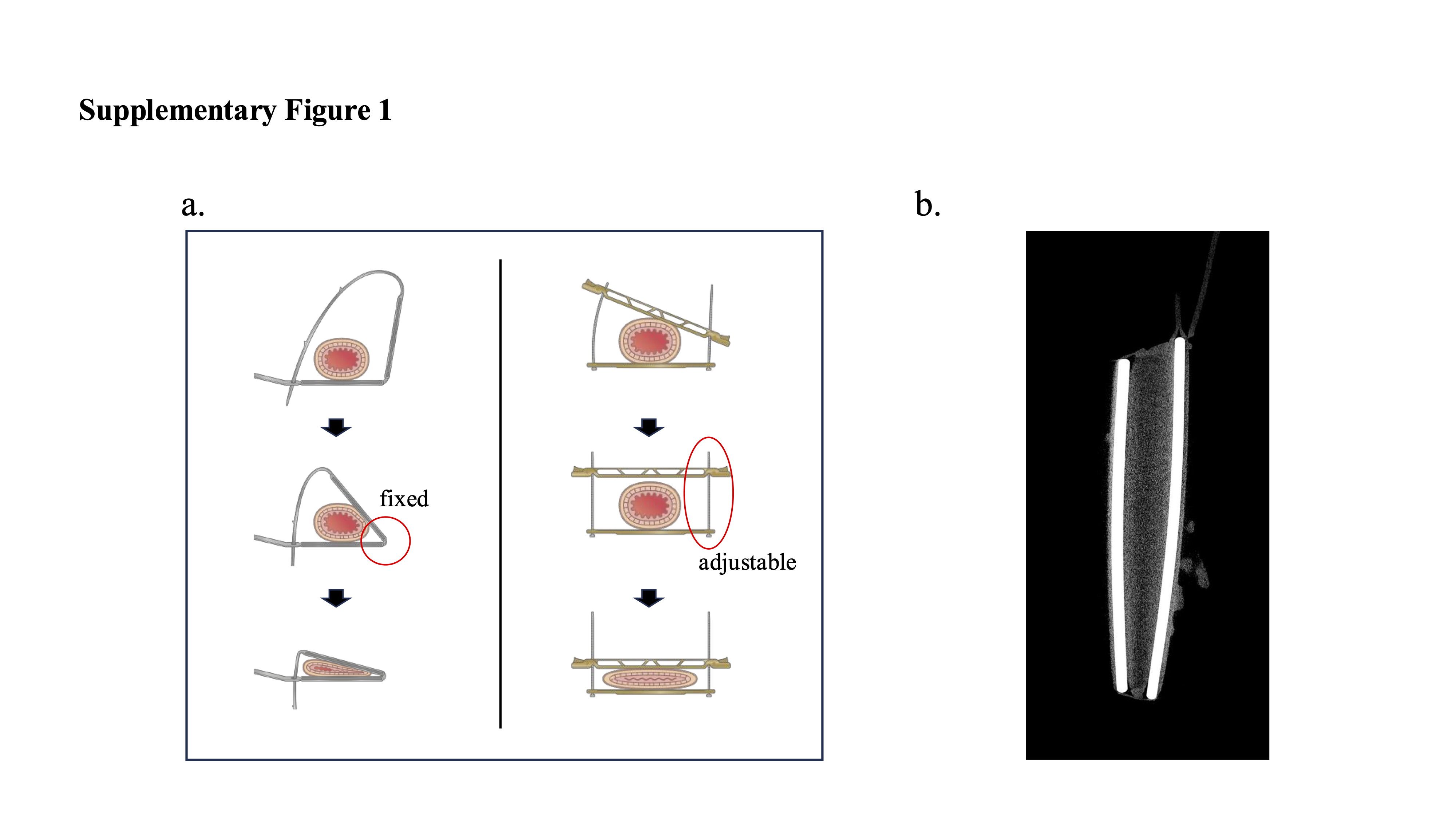

Supplement: Supplementary file 2 — Supplementary Material 2 [file 41598_2024_75103_MOESM2_ESM.tiff]
