# Supplementary material for: Development of novel rectal/uterine clamping device
Source: Sci Rep. 2024 Oct 3;14:22960. doi: 10.1038/s41598-024-75103-y (PMC11450202; doi:10.1038/s41598-024-75103-y)
Supplement: Supplementary file 3 — Supplementary Material 3 [file 41598_2024_75103_MOESM3_ESM.docx]

**Supplementary Figure 1**

**a,** Difference between the belt-type clamper and the New clamper. **b,** The bars of the belt-type clamper spread from the hinge side to the distal side, and the bars are deformed due to the strong force required to clamp.
